# Self-Association Behavior of Cell Membrane-Inspired Amphiphilic Random Copolymers in Water

**DOI:** 10.3390/polym11020327

**Published:** 2019-02-13

**Authors:** Maho Ohshio, Kazuhiko Ishihara, Shin-ichi Yusa

**Affiliations:** 1 Department of Applied Chemistry, Graduate School of Engineering, University of Hyogo, 2167 Shosha, Himeji, Hyogo 671-2280, Japan; mh11milk@gmail.com; 2 Department of Materials Engineering, School of Engineering, The University of Tokyo, 7-3-1 Hongo, Bunkyo-ku Tokyo 113-8656, Japan; ishihara@mpc.t.u-tokyo.ac.jp

**Keywords:** phosphorylcholine, amphiphilic copolymer, RAFT, hydrophobic interaction

## Abstract

Water-soluble and amphiphilic random copolymers (P(MPC/DMA*_x_*)) composed of hydrophilic 2-methacryloyloxyethyl phosphorylcholine (MPC) and hydrophobic *n*-dodecyl methacrylate (DMA) were prepared via reversible addition-fragmentation chain transfer (RAFT) controlled radical polymerization. The compositions of DMA unit (*x*) in the copolymer were in the range of 0 to 38 unit mol %. The degree of polymerization of P(MPC/DMA*_x_*) was adjusted to about 200. Since the monomer reactivity ratios of MPC and DMA are 1.01 and 1.00, respectively, ideal free radical copolymerization occurred. In aqueous solutions, interpolymer aggregation occurred due to the hydrophobic pendant *n*-dodecyl groups. The aggregation number (*N*_agg_) increased with an increasing *x*. The mobilities of the DMA and MPC pendant groups in aqueous solutions were restricted, as confirmed by ^1^H NMR relaxation time measurements, because a part of the MPC units were trapped in the hydrophobic microdomain formed from the pendant *n*-dodecyl groups. The polarity of the hydrophobic microdomain formed from P(MPC/DMA_38_) in water was similar to that of ethyl acetate according to fluorescence probe experiments. No specific interactions were found in water between P(MPC/DMA*_x_*) and bovine serum albumin because the surface of the interpolymer aggregates contained only hydrophilic MPC units.

## 1. Introduction

From the viewpoint of the biomedical applications of any materials, preparation of biocompatible surfaces must be considered. As one of the acceptable concepts for biocompatible surface, mimicking the cell membrane structure is widely recognized. Cell membranes have phospholipid bilayer membrane structures with a ca. 5 nm thickness, by self-organization of phospholipid molecules by hydrophobic interactions between longer alkyl groups combined with phospholipid polar groups [1]. The outer surface of the cellular membrane is covered with electrically neutral phosphorylcholine groups and these phosphorylcholine groups can suppress undesirable biological reactions from the viewpoint of medical implants and devices, such as blood coagulation [2].

Research on artificially preparing cell membranes is actively pursued by countless groups. It has been reported that when natural phospholipids are dispersed in water, they readily form vesicles with a lipid bilayer membrane structure [3]. However, lipid bilayer structures formed from low molecular weight phospholipids have poor physicochemical and biochemical stability [4]. Therefore, it would be interesting to create stable cell membrane-like artificial structures using polymers bearing pendant phosphorylcholine groups.

The polymers composed of 2-methacryloyloxyethyl phosphorylcholine (MPC) units having a pendant phosphorylcholine group, shows excellent biocompatibility; therefore, it can be used as a biocompatible material [5]. Polymers of various structures containing MPC have been reported: e.g., random copolymers [6], block copolymers [7], graft copolymers [8], and terminal group-functionalized polymers [9]. These MPC-containing copolymers have found application in various devices, such as biosensors [10,11], biochips [12], and bioimaging tools [13,14].

In water, an amphipathic random copolymer will sometimes form a hydrophobic microdomain by intrapolymer self-organization of hydrophobic moieties. This phenomenon is dependent on its chemical structure and the composition of its hydrophilic/hydrophobic groups [15]. The hydrophilic groups surround the hydrophobic microdomain, called a unimolecular micelle (unimer micelle). These unimer micelles are unlike conventional low molecular-weight surfactants in that they do not have a critical micelle concentration (cmc). Since hydrophobic molecules can be encapsulated into the hydrophobic microdomain, such a material is expected to find applications in drug delivery systems (DDS). Morishima et al [16]. reported that random copolymers composed of hydrophilic 2-acrylamido-2-methylpropanesulfonate and hydrophobic *N*-(*n*-dodecyl)methacrylamide form unimer micelles in water. The association state of the amphiphilic random copolymer was dependent on several conditions, such as the solvent, chemical additives, and external physical stimuli including pH and temperature. Recently, Terashima and Sawamoto et al. [17] reported the preparation of controlled-structured amphiphilic random copolymers composed from hydrophilic poly(ethylene glycol) methyl ether methacrylate (PEGMA) and hydrophobic alkyl methacrylates of varying alkyl chain lengths, as well as compositions via a controlled radical polymerization method using a ruthenium-complex catalyst. In water, the amphiphilic random copolymers containing 10–40 mol % of *n*-dodecyl methacrylate (DMA) formed unimer micelles with a DMA core and PEGMA shell due to the hydrophobic interactions within a single polymer chain. The radii of the unimer micelles decreased with increasing DMA content. When the DMA content exceeded 50 mol %, the polymer chains formed interpolymer aggregates. Compared to DMA-containing unimer micelles, amphiphilic random copolymers containing *n*-octadecyl methacrylate with longer pendant alkyl chains formed more compact unimer micelles. Amphiphilic random acrylate-type copolymers bearing poly(ethylene glycol) (PEG) and *n*-octadecyl acrylate (ODA) have also been reported [18]. The random copolymers containing 25–50 mol % ODA formed small unimer micelles with a radius of 4.8–8.8 nm in water. In the case of the MPC polymers, water-soluble MPC polymer having *n*-butyl methacrylate (BMA) units were synthesized, and their solubilizing state in the aqueous medium has been examined [19]. The polymer formed aggregates and they could solubilize hydrophobic compounds in the aggregate [20].

**Figure 1:**
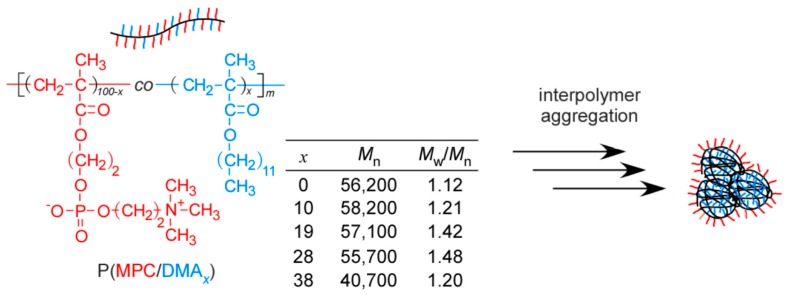
Chemical structure of the random copolymer (P(MPC/DMA*_x_*)) and a conceptual illustration of interpolymer aggregation.

In the current study, we focused on the fact that the phospholipids in cell membranes are composed of hydrophilic phosphorylcholine and hydrophobic alkyl groups. Amphiphilic random copolymers (P(MPC/DMA*_x_*)) composed of hydrophilic MPC and hydrophobic DMA were prepared via controlled reversible addition-fragmentation chain transfer (RAFT) radical polymerization (Figure 1). The subscript, *x*, in P(MPC/DMA*_x_*) denotes the DMA content (0–38 mol %). The degree of polymerization (DP) of P(MPC/DMA*_x_*) was 185–200 with narrow molecular weight distribution (*M*_w_/*M*_n_). P(MPC/DMA*_x_*) formed interpolymer aggregates composed of a hydrophobic microdomain formed from the pendant *n*-dodecyl groups, which was covered with the hydrophilic phosphorylcholine groups in water. The interpolymer aggregates showed a protein antifouling property, because the surface of the aggregates was covered with biocompatible phosphorylcholine groups. The resulting interpolymer aggregates formed from P(MPC/DMA*_x_*) in water were characterized using ^1^H NMR relaxation time studies, dynamic light scattering (DLS), static light scattering (SLS), transmission electron microscopy (TEM), and fluorescence probe techniques.

## 2. Experimental Section

### 2.1. Materials

2-Methacryloyloxyethyl phosphorylcholine (MPC, NOF, Tokyo, Japan) was purified according to a previously reported method prior to use [21]. *n*-Dodecyl methacrylate (DMA, 95%, Wako Pure Chemicals, Osaka, Japan) was passed through a basic alumina column to remove the inhibitor. 2,2’-Azobis(isobutyronitrile) (AIBN, Wako Pure Chemicals) and pyrene (97%, Wako Pure Chemicals) were purified by recrystallization from methanol. 4-Cyanopentanoic acid dithiobenzoate (CPD) was prepared according to the literature [22]. Ethanol and tetrahydrofuran (THF) were dried over molecular sieves 4 Å for one day and purified by distillation. Water was purified with an ion-exchange system. Bovine serum albumin (BSA, pH 5.0–5.6 buffer solution, Wako Pure Chemical) was used without further purification. All other reagents were used as received.

### 2.2. Preparation of P(MPC/DMA_x_)

A typical procedure for RAFT random copolymerization (Figure S1) to prepare the random copolymer with the content of the DMA unit (*x*) was 10 mol % was performed as follows: MPC (3.67 g, 12.4 mmol), DMA (353 mg, 1.39 mmol), CPD (19.1 mg, 6.85 × 10^−2^ mmol), and AIBN (4.64 mg, 2.85 × 10^−2^ mmol) were dissolved in a mixed solvent of THF and ethanol (13.8 mL, 1/1, *v*/*v*). The solution was deoxygenated by purging with argon gas for 30 min. Polymerization was carried out at 60 °C for 16 h under an argon atmosphere. The reaction mixture was dialyzed against methanol for 24 h and pure water for 24 h. The random copolymer ((P(MPC/DMA_10_)) was recovered using a freeze-drying technique (2.54 g, 63.3%). The number-average molecular weight calculated from NMR (*M*_n_(NMR)) was 5.78 × 10^4^, as estimated from comparing the integral intensity ratio of the ^1^H NMR peaks attributed to the terminal phenyl protons at 7.44–7.90 ppm and the MPC pendant methylene protons at 3.74 ppm. *x* were confirmed to be 10 mol % by comparing the integral intensity ratio of the ^1^H NMR peaks attributed to the MPC pendant methylene protons at 3.74 ppm and the DMA pendant methylene protons at 4.08 ppm. The number-average molecular weight estimated from gel-permeation chromatography (GPC) (*M*_n_(GPC)) and the molecular weight distribution (*M*_w_/*M*_n_) were 5.78 × 10^4^ and 1.21, respectively. Other random copolymers with *x* = 0, 19, 28, and 38 mol % were prepared in the same manner. All of the copolymers were characterized in a similar manner and the data is summarized in Table 1.

To study the relationship between monomer conversion and polymerization time, we performed the flowing experiment: MPC (221 mg, 0.749 mmol), DMA (191 mg, 0.752 mmol), CPD (2.17 mg, 7.77 × 10^−3^ mmol), and AIBN (0.510 mg, 3.00 × 10^−3^ mmol) were dissolved in a mixed solvent of THF and ethanol (1.3 mL, 1/1, *v*/*v*). Ethanol-*d*_6_ (0.2 mL) was then added. The reaction mixture was divided into multiple NMR tubes and argon gas was bubbled through the mixtures for 30 min. Polymerization was performed at 60 °C under an argon atmosphere for various polymerization times. Polymerization was terminated by cooling the NMR tube in an ice bath and the ^1^H NMR signals were monitored as a function of polymerization time. The conversions of MPC and DMA were separately monitored using the integral intensity ratio of the vinyl protons at 6.10 and 6.04 ppm, respectively (Figure S2).

### 2.3. Characterization of Polymers and Their Aggregates

^1^H NMR measurements were obtained using a Bruker (Billerica, MA, USA) DRX-500 spectrometer. ^1^H NMR sample solutions were prepared in D_2_O, methanol-*d*_4_, and ethanol-*d*_6_ as appropriate. The ^1^H NMR spin-spin relaxation time (*T*_2_) was determined using the Carr-Purcell-Meiboom-Gill (CPMG) method: Echo peak intensities of the 180° pulse were measured at 16 different numbers [23]. GPC measurements were performed using a Jasco RI-2031 plus RI detector equipped with a Jasco PU-2080 pump and a Shodex (Tokyo, Japan) OHpak SB-G and SB-804HQ column. A 0.3 M Na_2_SO_4_ aqueous solution containing 0.5 M acetic acid was used as the eluent at a flow rate of 0.6 mL/min at 40 °C. *M*_n_(GPC) and *M*_w_/*M*_n_ were calibrated using poly(2-vinylpyridine) standards. Dynamic light scattering (DLS) measurements were performed using a Malvern (Malvern, UK) Zetasizer nano ZS equipped with a He–Ne laser (4 mW at 632.8 nm) at 25 °C. All samples for light scattering experiments were filtered through a 0.2 μm pore-size membrane. The obtained data was analyzed using the Malvern Zetasizer Software package v7.11. Static scattering light (SLS) measurements were performed using an Otsuka Electronic Photal (Osaka, Japan) DLD-7000 at 25 °C. A He–Ne laser (10.0 mW at 632.8 nm) was used as the light source. The weight-average molecular weight (*M*_w_), radius of gyration (*R*_g_), and the second virial coefficient (*A*_2_) were estimated from Zimm plots. Values of d*_n_*/d*_C_*_p_ at 633 nm were determined with an Otsuka Electronics Photal DRM-3000 differential refractometer at 25 °C. Transmission electron microscopy (TEM) was performed using a Jeol (Tokyo, Japan) JEM-2100 with an accelerating voltage of 200 kV. Samples for TEM observation were prepared by placing one drop of the aqueous solution on a copper grid coated with thin films of Formvar and carbon. Excess water was blotted using filter paper. The samples were stained with sodium phosphotungstate and dried under vacuum. Fluorescence measurements were performed using a Hitachi (Tokyo, Japan) F-2500 fluorescence spectrophotometer. The polymer was dissolved in a pyrene-saturated aqueous solution (6.0 × 10^−7^ M) at *C*_p_ = 5 g/L. The solution was excited at 334 nm, and the excitation and emission slit widths were maintained at 20 and 2.5 nm, respectively.

## 3. Results and Discussion

### 3.1. Preparation of P(MPC/DMA_x_)

The conversions of MPC and DMA monomers at various polymerization times were monitored using ^1^H NMR during the preparation of P(MPC/DMA_50_). The conversions of MPC and DMA were estimated from the integral intensity ratios of MPC (at 5.60 and 6.10 ppm) and DMA vinyl protons (at 5.54 and 6.04 ppm) using the water peak as an internal standard (Figure S2). The time-conversion profiles of MPC and DMA were nearly identical (Figure S3). This observation indicated that the consumption rates of MPC and DMA during the copolymerization reaction were similar. Induction periods of about 60 min for MPC and DMA were observed; thereafter, both conversions reached about 90% after 520 min of polymerization. The first order kinetic plots of MPC and DMA were linear, suggesting that the concentration of propagating radicals was constant during the entire random copolymerization process. In order to determine the monomer reactivity ratios of MPC (*r*_MPC_) and DMA (*r*_DMA_), the DMA content (*x*) in P(MPC/DMA*_x_*) was plotted against the feed DMA ratio (Figure S4a). The DMA composition in the random copolymer was nearly identical to that of the feed DMA ratio. The *r*_MPC_ and *r*_DMA_ values were 1.01 and 1.00, respectively, as obtained from the Fineman-Ross plot (Figure S4b). These observations suggest that the MPC and DMA distribution in P(MPC/DMA*_x_*) should be completely random. The GPC elution curves for P(MPC/DMA*_x_*) were unimodal with *M*_w_/*M*_n_ (Figure S5) and the theoretical number-average molecular weight (*M*_n_(theory)) can be estimated from the following equation:

(1) $$M_{n}\left(\mathrm{theory}\right)=\;\frac{{\left[M\right]}_{0}}{{\left[\mathrm{CTA}\right]}_{0}}\times \frac{p}{100}\times M_{M}+M_{\mathrm{CTA}}$$

where [M]_0_ is the initial total monomer concentration of MPC and DMA, [CTA]_0_ is the initial CTA concentration, *p* is the average percent conversion of the monomer, M_M_ is the average-molecular weight of two monomers, and M_CTA_ is the molecular weight of CTA. The DMA composition, *M*_n_(theory), *M*_n_(NMR), *M*_n_(GPC) DP, and *M*_w_/*M*_n_ for P(MPC/DMA*_x_*) are summarized in Table 1. The *M*_n_(theory) and *M*_n_(NMR) values were similar and the *M*_w_/*M*_n_ ratio was narrower than 1.48. These observations suggest that the random copolymerization proceeded in accordance with a “living” mechanism.

**Figure 2:**
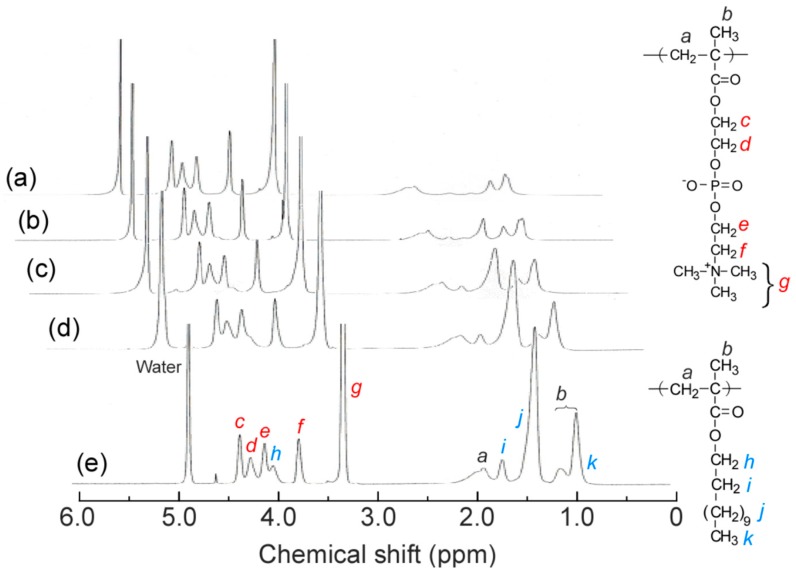
^1^H NMR spectra for P(MPC/DMA*_x_*) in methanol-*d*_4_: *x* = 0 (**a**), 10 (**b**), 19 (**c**), 28 (**d**), and 38 mol % (**e**).

**Figure 3:**
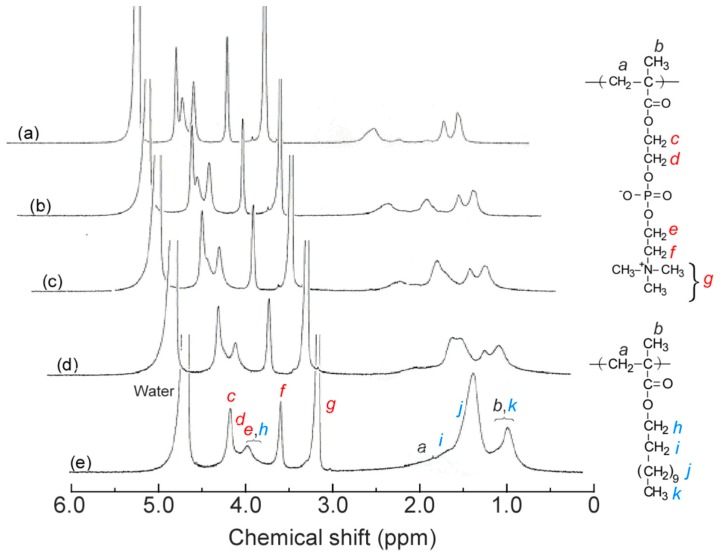
^1^H NMR spectra for P(MPC/DMA*_x_*) in D_2_O containing 0.1 M NaCl: *x* = 0 (**a**), 10 (**b**), 19 (**c**), 28 (**d**), and 38 mol % (**e**).

^1^H NMR measurements were performed for P(MPC/DMA*_x_*) (*x* = 0–38 mol %) in methanol-*d*_4_ (Figure 2). The accurate value of *x* was calculated from the integral intensity ratios of the pendant methylene protons in MPC unit at 3.74 ppm (*f*) and the pendant methylene protons in DMA unit at 4.08 ppm (*h*). ^1^H NMR measurements were also performed for P(MPC/DMA*_x_*) in D_2_O containing 0.1 M NaCl (Figure 3). The pendant methylene protons in DMA at 4.00 ppm broadened with increasing *x* because the motion of DMA was restricted. The pendant hydrophobic *n*-dodecyl groups aggregated in D_2_O containing 0.1 M NaCl to form a hydrophobic microdomain.

### 3.2. Self-Association Behavior of P(MPC/DMA_x_)

**Figure 4:**
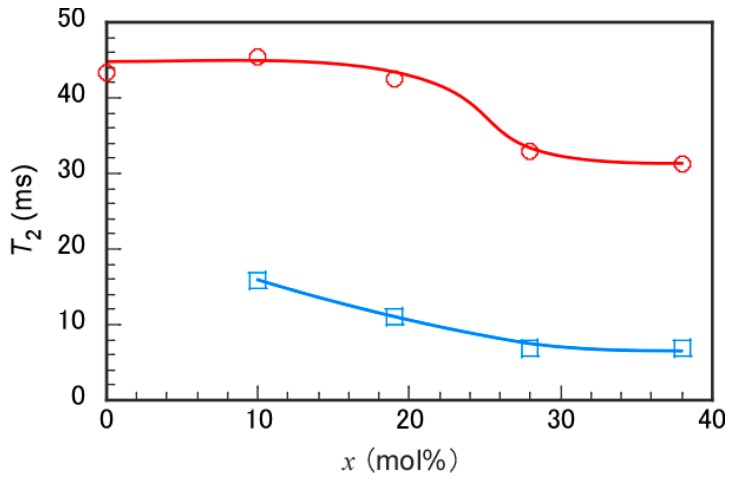
Spin-spin relaxation time (*T*_2_) of the MPC pendant methyl protons at 3.21 ppm (〇) and the DMA pendant methylene protons near 1.5 ppm (□) as a function of the DMA content (*x*) in P(MPC/DMA*_x_*) in D_2_O containing 0.1 M NaCl.

To study proton mobility in P(MPC/DMA*_x_*), the spin-spin relaxation time (*T*_2_) was measured in D_2_O containing 0.1 M NaCl (Figure 4). The *T*_2_ values were estimated from the pendant methyl protons in MPC units at 3.21 ppm and in DMA units at 1.2–1.6 ppm. *T*_2_ decreases with decreasing proton mobility [24]. The *T*_2_ values for DMA units were always smaller than those for MPC units, which suggested that the motion of DMA units was more restricted than that of MPC units. The pendant *n*-dodecyl groups may form a hydrophobic microdomain and the pendant phosphorylcholine groups may surround the surface of the microdomain. When *x* exceeded 19 mol %, the *T*_2_ values for MPC unit decreased gradually. This observation suggested that some hydrophilic MPC units may be incorporated into the hydrophobic microdomains due to the random distributions of MPC and DMA units within a single polymer chain.

**Figure 5:**
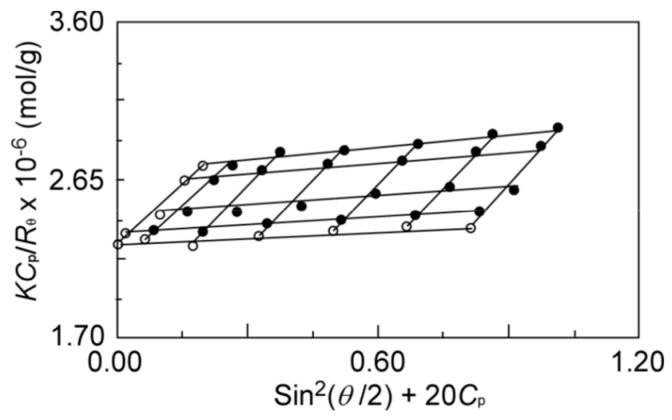
A representative Zimm plot of P(MPC/DMA_19_) in 0.1 M NaCl aqueous solution.

To characterize the aggregates formed by P(MPC/DMA*_x_*), SLS measurements were performed in 0.1 M NaCl aqueous solutions. Figure 5 shows a representative Zimm plot of P(MPC/DMA_19_). From the Zimm plots (Figure S6), the apparent weight-average molecular weight (*M*_w_), the second virial coefficient (*A*_2_), and the *z*-average radius of gyration (*R*_g_) were estimated (Table 2).

The aggregation number (*N*_agg_) was calculated from the apparent *M*_w_ estimated from SLS and the *M*_w_ of the single polymer chain. The *M*_w_ value of a single polymer chain was calculated from the *M*_n_(NMR) and *M*_w_/*M*_n_ values. The *N*_agg_ value of a PMPC homopolymer was 1.1, indicating that PMPC dissolved in 0.1 M NaCl in a unimer state. The *N*_agg_ of P(MPC/DMA*_x_*) increased with increasing *x* because the hydrophobic interactions of the surface *n*-dodecyl groups on the intrapolymer aggregates induced interpolymer aggregation. The hydrophobic *n*-dodecyl groups in P(MPC/DMA*_x_*) may aggregate within a single polymer chain, and the surface is then covered by the hydrophilic phosphorylcholine groups. Since the phosphorylcholine groups cannot cover the entire surface of the aggregate as *x* increases, some hydrophobic *n*-dodecyl groups become exposed on the surface. The *A*_2_ value can be used as an indicator of the affinity of a polymer with the solvent [25]. With increasing hydrophobic *n*-dodecyl group content in the polymer chain, the solubility of the polymer in water decreased. Therefore, the *A*_2_ value decreased with increasing *x*. The *R*_g_/*R*_h_ ratio is a structure sensitive parameter that provides information on the morphology, density, and size distribution of the aggregates. According to the literature, the *R*_g_/*R*_h_ of a rigid sphere is 0.775, a sphere is 1, and a rod is greater than 2 [26]. Since the *R*_g_/*R*_h_ of the random copolymers with *x* ≤ 28 mol % was relatively close to 1, these polymers may form spherical aggregates. However, the *R*_g_/*R*_h_ of P(MPC/DMA_38_) was large (=2.69), suggesting that the shape of these aggregates was not spherical or large polydispersity index (PDI).

**Figure 6:**
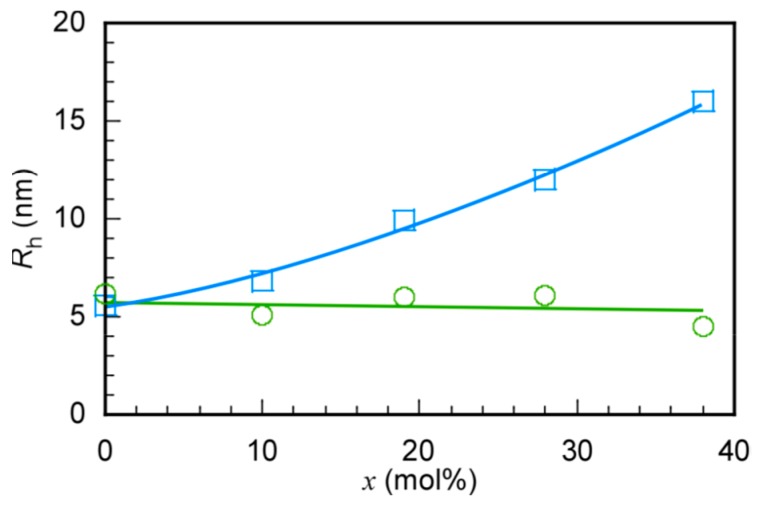
Hydrodynamic radius (*R*_h_) of P(MPC/DMA*_x_*) in 0.1 M NaCl aqueous solutions (□) and in methanol (〇) as a function of the DMA content in the copolymer (*x*).

DLS measurements of P(MPC/DMA*_x_*) were performed in 0.1 M NaCl aqueous (Figure S7) and methanol solutions (Figure S8). All *R*_h_ distributions were unimodal and the *R*_h_ values of P(MPC/DMA*_x_*) were plotted against *x* in both solutions (Figure 6). The *R*_h_ values of the PMPC homopolymer in 0.1 M NaCl aqueous and methanol were 5.6 and 6.2 nm, respectively. This observation indicated that the PMPC homopolymer can dissolve in a unimer state in water and methanol. The *R*_h_ values of P(MPC/DMA*_x_*) increased with increasing *x* in 0.1 M NaCl aqueous solutions. The *R*_h_ values increased because the *N*_agg_ increased. This was caused by interpolymer aggregation of the hydrophobic *n*-dodecyl groups. Although the PDI of the *x* ≤ 28 mol% copolymers were less than 0.26 in 0.1 M NaCl aqueous solutions, the PDI of P(MPC/DMA_38_) was broad (=0.42). This observation is consistent with the *R*_g_/*R*_h_ for P(MPC/DMA_38_). The PDI of P(MPC/DMA*_x_*) in methanol were less than 0.29 (Figure S8). The *R*_h_ values of P(MPC/DMA*_x_*) in methanol appeared to be independent of *x* and stayed within 4.5–6.1 nm, which was close to the *R*_h_ (=6.2 nm) of the PMPC homopolymer. These observations suggest that P(MPC/DMA*_x_*) can dissolve in methanol in a unimer state without interpolymer aggregates.

**Figure 7:**
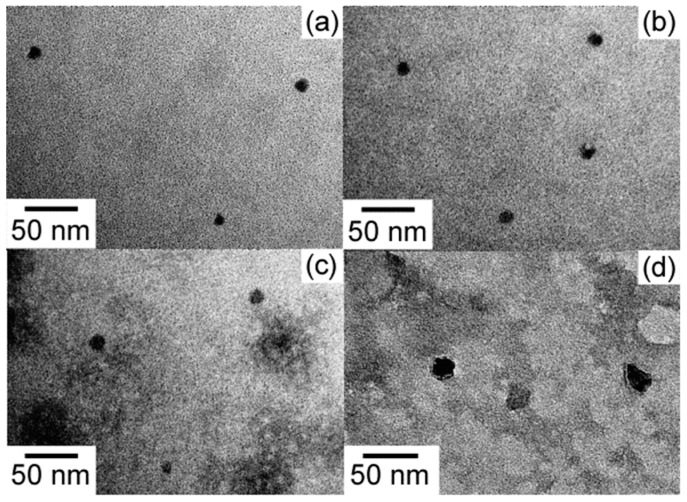
TEM images of P(MPC/DMA*_x_*) with *x* = 10 (**a**), 19 (**b**), 28 (**c**), and 38 mol % (**d**).

From the TEM images (Figure 7), it was confirmed that P(MPC/DMA*_x_*) formed aggregates. The radii (*R*_TEM_) estimated from TEM were smaller than the *R*_h_ values determined by DLS (Table 2), likely because the aggregates shrank during the drying process involved in TEM sample preparation.

**Figure 8:**
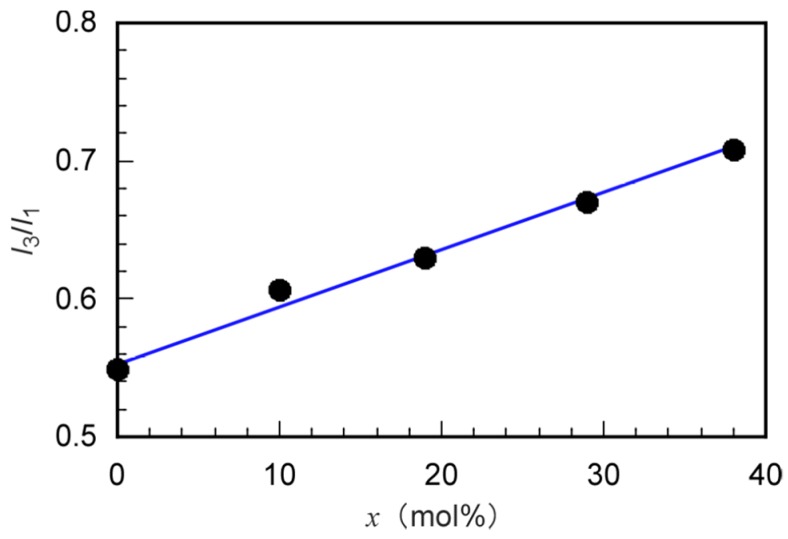
Pyrene fluorescence vibrionic peak intensity ratio (*I*_3_/*I*_1_) in the presence of P(MPC/DMA*_x_*) as a function of DMA content (*x*): *I*_3_ and *I*_1_ are the intensities of third and first vibrionic peaks in pyrene fluorescence, respectively.

The intensity ratio (*I*_3_/*I*_1_) of the first (*I*_1_) and the third vibrionic peaks (*I*_3_) of the fluorescence spectra of pyrene as a hydrophobic fluorescent probe depends on the microenvironmental polarity around the pyrene molecule [27]. While *I*_3_/*I*_1_ increases in a hydrophobic environment, the corollary decrease in a hydrophilic environment holds true. The fluorescence spectra of aqueous pyrene solutions without a polymer and the mixture of pyrene with P(MPC/DMA_38_) were compared (Figure S9). The *I*_3_/*I*_1_ ratios of pyrene fluorescence in the absence and presence of P(MPC/DMA_38_) were 0.55 and 0.71, respectively. *I*_3_/*I*_1_ was larger in the presence of P(MPC/DMA_38_) because pyrene was encapsulated into the hydrophobic environment formed from aggregation of the pendant *n*-dodecyl groups. We measured the polarity of the hydrophobic domains using *I*_3_/*I*_1_ (Figure 8). With increasing *x*, *I*_3_/*I*_1_ became larger due to increasing the hydrophobicity. The hydrophobicity of the microdomain became stronger with increasing *x* because the number of hydrophobic *n*-dodecyl groups forming a microdomain increased. The hydrophobicity within the microdomain (*I*_3_/*I*_1_ = 0.72) in P(MPC/DMA_38_) was similar to that of ethyl acetate. The *I*_3_/*I*_1_ of pyrene in ethyl acetate is 0.69 [27].

**Figure 9:**
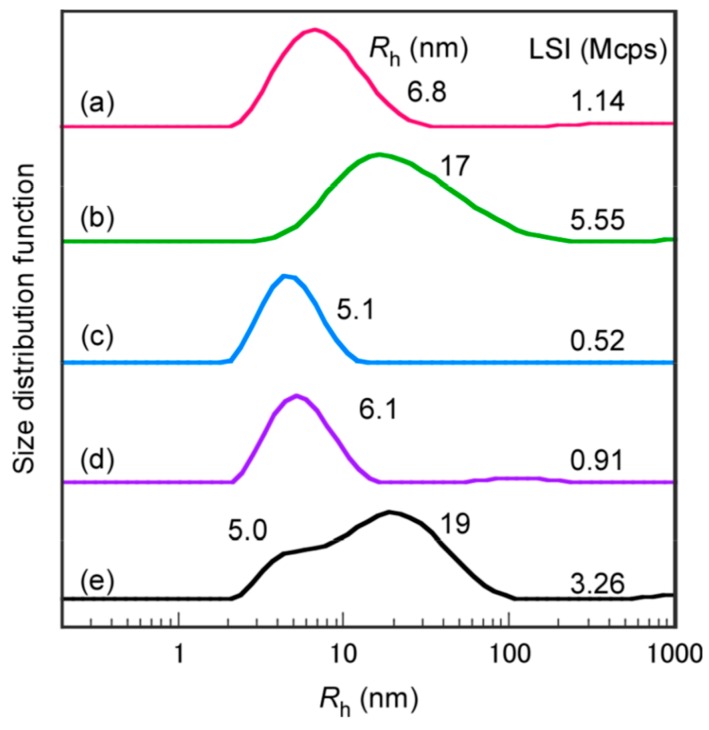
Hydrodynamic radius (*R*_h_) distributions and light scattering intensity (LSI) for (**a**) P(MPC/DMA_10_), (**b**) P(MPC/DMA_38_), (**c**) BSA, (**d**) mixture of P(MPC/DMA_10_) and BSA, and (**e**) mixture of P(MPC/DMA_38_) and BSA in phosphate buffered saline (PBS) at 25 °C. The concentrations of random copolymers and BSA were fixed at 5.0 g/L.

Generally, MPC polymers show protein adsorption resistance [5]; therefore, we studied the interaction between P(MPC/DMA*_x_*) (*x* = 10 and 38) and BSA protein in phosphate buffered saline (PBS) at 25 °C using DLS. The *R*_h_ distributions of P(MPC/DMA_10_), BSA, and the mixture with the same concentrations of P(MPC/DMA_10_) with BSA were unimodal (Figure 9a,c,d). The *R*_h_ values of P(MPC/DMA_10_) and BSA were 6.8 and 5.1 nm, respectively. The *R*_h_ value of the mixture was 6.1 nm, which was similar to the *R*_h_ values before mixing. The light scattering intensities (LSIs) of P(MPC/DMA_10_) and BSA in PBS were 1.14 and 0.52 Mcps, respectively. After mixing, the LSI became 0.91 Mcps. These observations suggest that there was no interaction between P(MPC/DMA_10_) and BSA in PBS because the *R*_h_ and LSI of the mixture did not change greatly from the pre-mixing values. Since the aggregates of P(MPC/DMA_10_) were covered by hydrophilic phosphorylcholine groups, the aggregates showed protein antifouling properties. Similarly, the *R*_h_ distributions of P(MPC/DMA_38_), BSA, and the an equimolar mixture were measured with DLS (Figure 9b,c,e). The *R*_h_ of P(MPC/DMA_38_) (=17 nm) and the mixture (=5.0 and 19 nm) were unimodal with broad and bimodal distributions. The *R*_h_ distribution of the mixture may have overlapped those of P(MPC/DMA_38_) and BSA. The LSI of the mixture (=3.26 Mcps) was similar to that of P(MPC/DMA_38_) (=5.55 Mcps) and BSA (=0.52 Mcps) combined. These observations suggest that there was no significant interaction between P(MPC/DMA_38_) and BSA and they suppressed adsorption of BSA in PBS at 25 °C. In the case of immunoassay, the water-soluble and amphiphilic MPC polymer can be used as a blocking reagent instead of BSA and casein for preventing non-selective adsorption of un-target molecules [28]. When the polymer attached on the solid surface, polymer aggregate can dissociate and hydrophobic core portion can attach directly on the substrate. It makes string adsorption force and phosphorylcholine group covered with the substrate. P(MPC/DMA_38_) had these properties as well and may be applied for this purpose.

## 4. Conclusions

Amphiphilic random copolymers (P(MPC/DMA*_x_*, *x* = 0–38 mol %) with well-controlled structures were prepared from MPC and DMA (0–38 mol %) via RAFT radical polymerization. In aqueous solutions, P(MPC/DMA*_x_*) formed interpolymer aggregates composed of hydrophobic cores containing pendant *n*-dodecyl groups covered by hydrophilic phosphorylcholine groups. The *N*_agg_ and *R*_h_ of the interpolymer aggregates increased with increasing DMA content, *x*. Furthermore, the hydrophobicity of the domain formed from the DMA units increased and the motion of the *n*-dodecyl groups decreased with increasing *x*. The motion of the hydrophilic phosphorylcholine groups was also restricted with increasing *x* because a portion of the phosphorylcholine groups were trapped within the hydrophobic domain formed from the *n*-dodecyl groups. P(MPC/DMA_10_) and P(MPC/DMA_38_) were tested for protein antifouling properties using BSA in PBS. The aggregates could suppress protein adsorption because the surface was covered with phosphorylcholine groups. 

## Figures and Tables

**Table 1:** Conversion, Number-Average Molecular Weight (*M*_n_), Degree of Polymerization (DP), and Molecular Weight Distribution (*M*_w_/*M*_n_) of P(MPC/DMA*_x_*).

*x* (mol %)	Conversion (%)	*M*_n_(theory) × 10^−4^	*M*_n_(NMR) × 10^−4^	DP(NMR)	*M*_n_(GPC) × 10^−4^	*M*_w_/*M*_n_
0	94.7	5.62	5.91	200	2.93	1.12
10	99.7	5.82	5.78	197	2.81	1.21
19	99.5	5.71	5.60	194	3.27	1.42
28	99.8	5.65	5.57	195	3.14	1.48
38	74.2	4.07	5.21	185	2.57	1.20

**Table 2:** Dynamic and Static Light Scattering Data for P(MPC/DMA*_x_*) in 0.1 M NaCl Aqueous Solutions.

*x*	*M*_w_*^a^* × 10^−5^ (g/mol)	*R*_g_*^a^* (nm)	*R*_h_*^b^* (nm)	*R*_g_/*R*_h_	*R*_TEM_*^c^* (nm)	*N*_agg_*^d^*	*A*_2_*^a^* × 10^−5^ (cm^3^·g^−2^·mol)
0	0.60	8.08	5.6	1.44	-	1.1	27.1
10	2.49	7.90	6.80	1.16	5.45	3.6	26.9
19	4.47	11.3	9.90	1.14	6.94	5.2	2.11
28	7.82	21.0	11.8	1.78	7.58	8.4	1.59
38	8.76	45.9	17.6	2.61	14.0	13.7	1.40

*^a^* Estimated by SLS in 0.1 M NaCl aqueous solutions. *^b^* Estimated by DLS in 0.1 M NaCl aqueous solutions. *^c^* Estimated by TEM. *^d^* Aggregation number of a polymer micelle calculated from the *M*_w_ of the micelle determined by SLS and *M*_w_ of the corresponding unimers determined by *M*_n_(NMR) and *M*_w_/*M*_n_.

## Supplementary Materials

The following are available online at http://www.mdpi.com/2073-4360/11/2/327/s1: Figure S1. Synthesis of P(MPC/DMA*_x_*); Figure S2. ^1^H NMR spectra for RAFT random copolymerization of equimolar amounts of MPC and DMA (a) before and (b) after polymerization. The reaction was performed in a mixed solvent of THF and ethanol (1.3 mL, 1/1, v/v) with ethanol-*d*_6_ (0.2 mL); Figure S3. (a) Time-conversion and (b) the first-order kinetic plots for RAFT copolymerization of equimolar amounts of MPC (〇) and DMA (△): [M]_0_ and [M] were the monomer concentrations at polymerization time = 0 and the corresponding time, respectively; Figure S4. (a) Relationship between DMA content in the copolymer and feed DMA. (b) Relationship between *F*(*f*-1)/*f* and *F*^2^/*f*; *f* = *m*_MPC_/*m*_DMA_, *F* = [M_MPC_]_0_/[M_DMA_]_0_, where *m*_MPC_ and *m*_DMA_ are the composition of MPC and DMA in the copolymer, respectively, and [M_MPC_]_0_ and [M_DMA_]_0_ are the molar concentrations of MPC and DMA before polymerization, respectively; Determination of monomer reactivity ratio; Figure S5. GPC elution curves of P(MPC/DMA*_x_*) where *x* = (a) 0, (b) 10, (c) 19, (d) 28, and (e) 38 mol%; Figure S6. Zimm plots of P(MPC/DMA*_x_*) in 0.1 M NaCl aqueous solutions where *x* = (a) 0, (b) 10, (c) 19, (d) 28, and (e) 38 mol%; Figure S7. Hydrodynamic radius (*R*_h_) distributions and polydispersity index (PDI) for P(MPC/DMA*_x_*) in 0.1 M NaCl aqueous solutions at 25 °C where *x* = (a) 0, (b) 10, (c) 19, (d) 28, and (e) 38 mol%; Figure S8. Hydrodynamic radius (*R*_h_) distributions and polydispersity index (PDI) for P(MPC/DMA*_x_*) in methanol at 25 °C where *x* = (a) 0, (b) 10, (c) 19, (d) 28, and (e) 38 mol%; Figure S9. Fluorescence spectra of pyrene in the absence (---) and presence (—) of P(PMPC/DMA_38_) in 0.1 M NaCl aqueous solutions excited at 334 nm. The excitation and emission slit widths were fixed at 20 and 5.0 nm, respectively.
