# Supplementary material for: Self-Association Behavior of Cell Membrane-Inspired Amphiphilic Random Copolymers in Water
Source: Polymers (Basel). 2019 Feb 13;11(2):327. doi: 10.3390/polym11020327 (PMC6419178; doi:10.3390/polym11020327)
Supplement: Supplementary file 1 [file polymers-11-00327-s001.pdf]

Supporting Information

Self-association Behavior of Cell Membrane-Inspired Amphiphilic Random Copolymers in Water

Maho Ohshio[1], Kazuhiko Ishihara[2], Shin-ichi Yusa[1,*]

[1]Department of Applied Chemistry, Graduate School of Engineering, University of Hyogo, 2167 Shosha, Himeji, Hyogo 671-2280, Japan

[2]Department of Materials Engineering, School of Engineering, The University of Tokyo, 7-3-1 Hongo, Bunkyo-ku Tokyo 113-8656, Japan

* Correspondence: yusa@eng.u-hyogo.ac.jp; Tel: +81-79-267-4954; Fax: +81-79-266-8868

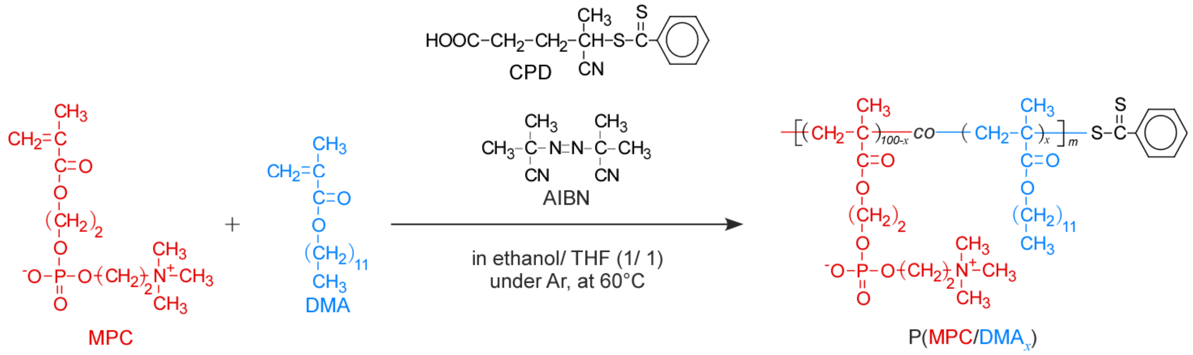

Figure S1. Synthesis of P(MPC/DMA$_x$).

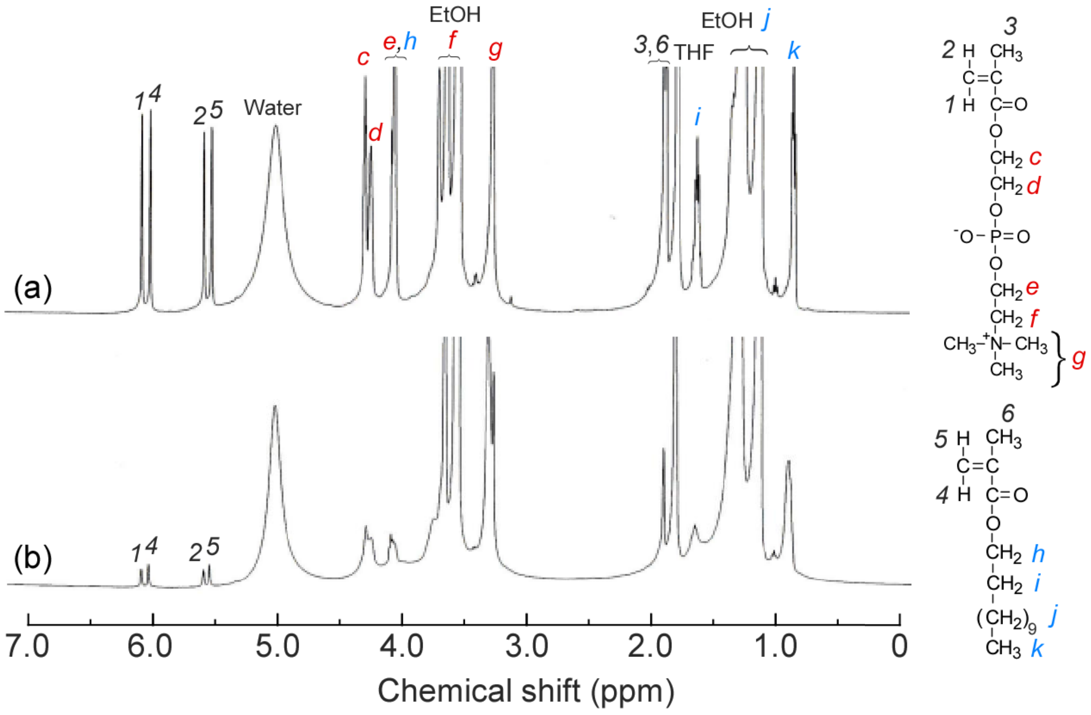

Figure S2. ^{1}H NMR spectra for RAFT random copolymerization of equimolar amounts of MPC and DMA (a) before and (b) after polymerization. The reaction was performed in a mixed solvent of THF and ethanol (1.3 mL, 1/1, v/v) with ethanol-d_6 (0.2 mL).

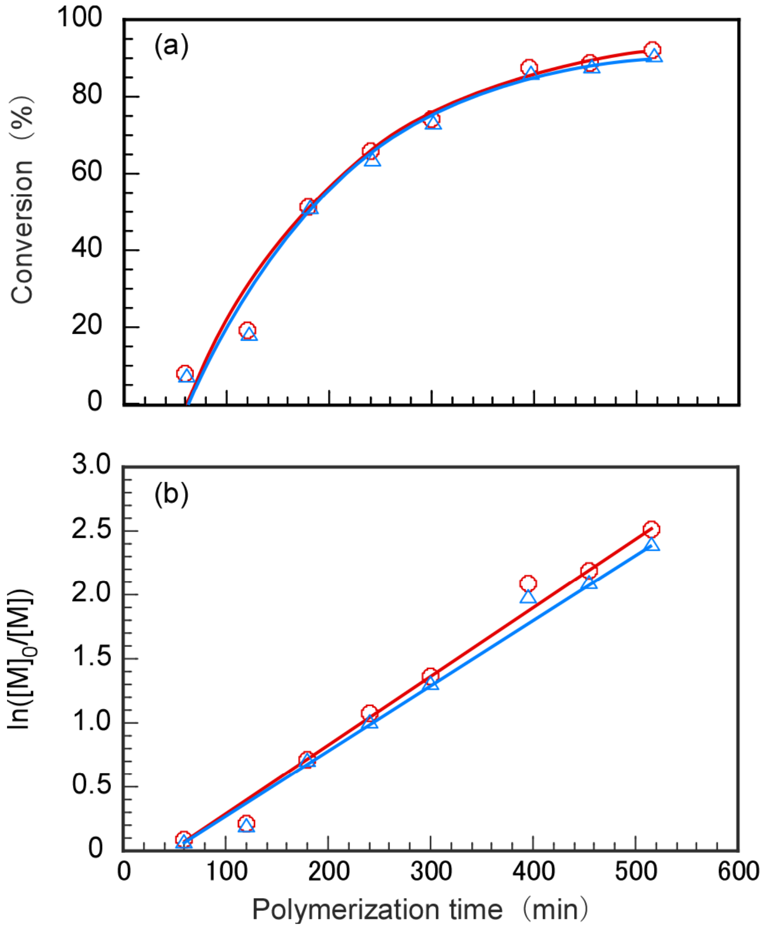

Figure S3. (a) Time-conversion and (b) the first-order kinetic plots for RAFT copolymerization of equimolar amounts of MPC (○) and DMA (△): $[M]_0$ and [M] were the monomer concentrations at polymerization time = 0 and the corresponding time, respectively.

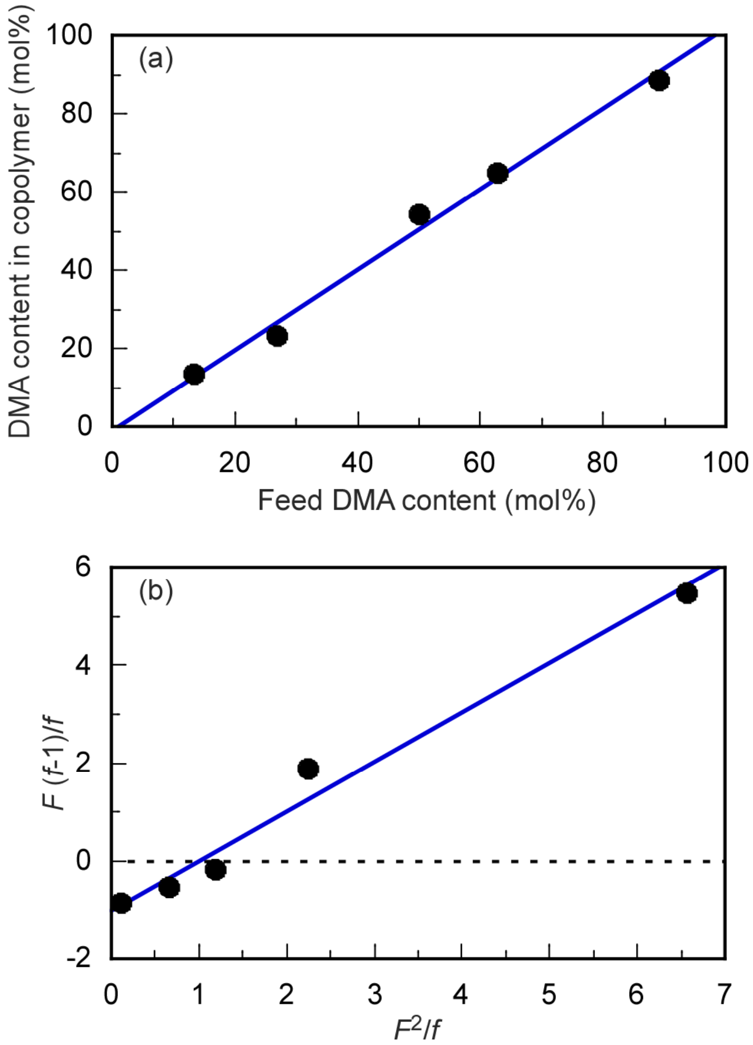

Figure S4. (a) Relationship between DMA content in the copolymer and feed DMA. (b) Relationship between $F(f\text{-}1)/f$ and F^2/f; $f = m_{MPC}/m_{DMA}$, $F = [M_{MPC}]_0/[M_{DMA}]_0$, where m_{MPC} and m_{DMA} are the composition of MPC and DMA in the copolymer, respectively, and $[M_{MPC}]_0$ and $[M_{DMA}]_0$ are the molar concentrations of MPC and DMA before polymerization, respectively.

Determination of monomer reactivity ratio.

MPC, DMA, CPD, and AIBN were dissolved in a mixed solvent of THF and ethanol (1/1, v / v) and the feed ratio of DMA was changed from 10 to 90 mol%: ([MPC] + [DMA]): [CPD]: [AIBN] = 200: 1: 0.4. Ethanol-d_6 was added and the solutions were transferred to NMR

tubes. The reaction mixtures were deoxygenated by purging with argon gas for 30 min. RAFT random copolymerization was performed at 60 °C for 100 min under an argon atmosphere. Polymerization was stopped with an ice bath while the total monomer conversion was less than 18%. The content of DMA in the random copolymer was estimated from the conversion of DMA estimated from ^{1}H NMR. The conversion of DMA was estimated by the integral intensity ratio of vinyl protons at 6.04 ppm (Figure S4a).

The ratio ($m_{\mathrm{MPC}}/m_{\mathrm{DMA}} = f$) of the MPC and DMA content in the copolymer obtained from random copolymerization can be represented by the following copolymer composition formula:

$$\frac{m_{\mathrm{MPC}}}{m_{\mathrm{DMA}}} = \frac{[M_{\mathrm{MPC}}]_0}{[M_{\mathrm{DMA}}]_0} \times \frac{r_{\mathrm{MPC}}[M_{\mathrm{MPC}}]_0 + [M_{\mathrm{DMA}}]_0}{[M_{\mathrm{MPC}}]_0 + r_{\mathrm{DMA}}[M_{\mathrm{DMA}}]_0} \tag{S1}$$

where m_{MPC} and m_{DMA} are the molar contents of MPC and DMA in the random copolymer, respectively, $[M_{\mathrm{MPC}}]_0$ and $[M_{\mathrm{DMA}}]_0$ are the molar concentration of MPC and DMA monomers before polymerization, respectively, and r_{MPC} and r_{DMA} are the monomer reactivity ratios of MPC and DMA, respectively. Equation S1 can be transformed to the Fineman-Ross equation form:

$$\frac{F(f-1)}{f} = \frac{r_{\mathrm{MPC}}F^2}{f} - r_{\mathrm{DMA}} \tag{S2}$$

where F (= $[M_{MPC}]_0/[M_{DMA}]_0$) is the feed ratio of MPC and DMA before polymerization. The resulting Fineman-Ross plot is presented in Figure S4b. r_{MPC} and r_{DMA} were estimated from the slope and intercept, respectively. The r_{MPC} and r_{DMA} values were 1.01 and 1.00, respectively.

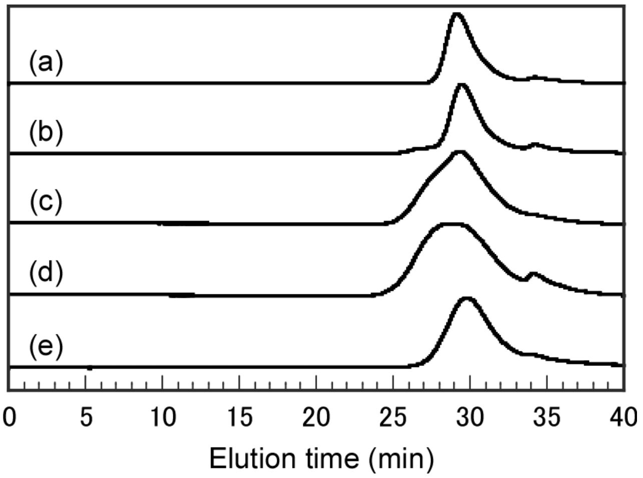

Figure S5. GPC elution curves of P(MPC/DMA$_x$) where x = (a) 0, (b) 10, (c) 19, (d) 28, and (e) 38 mol%.

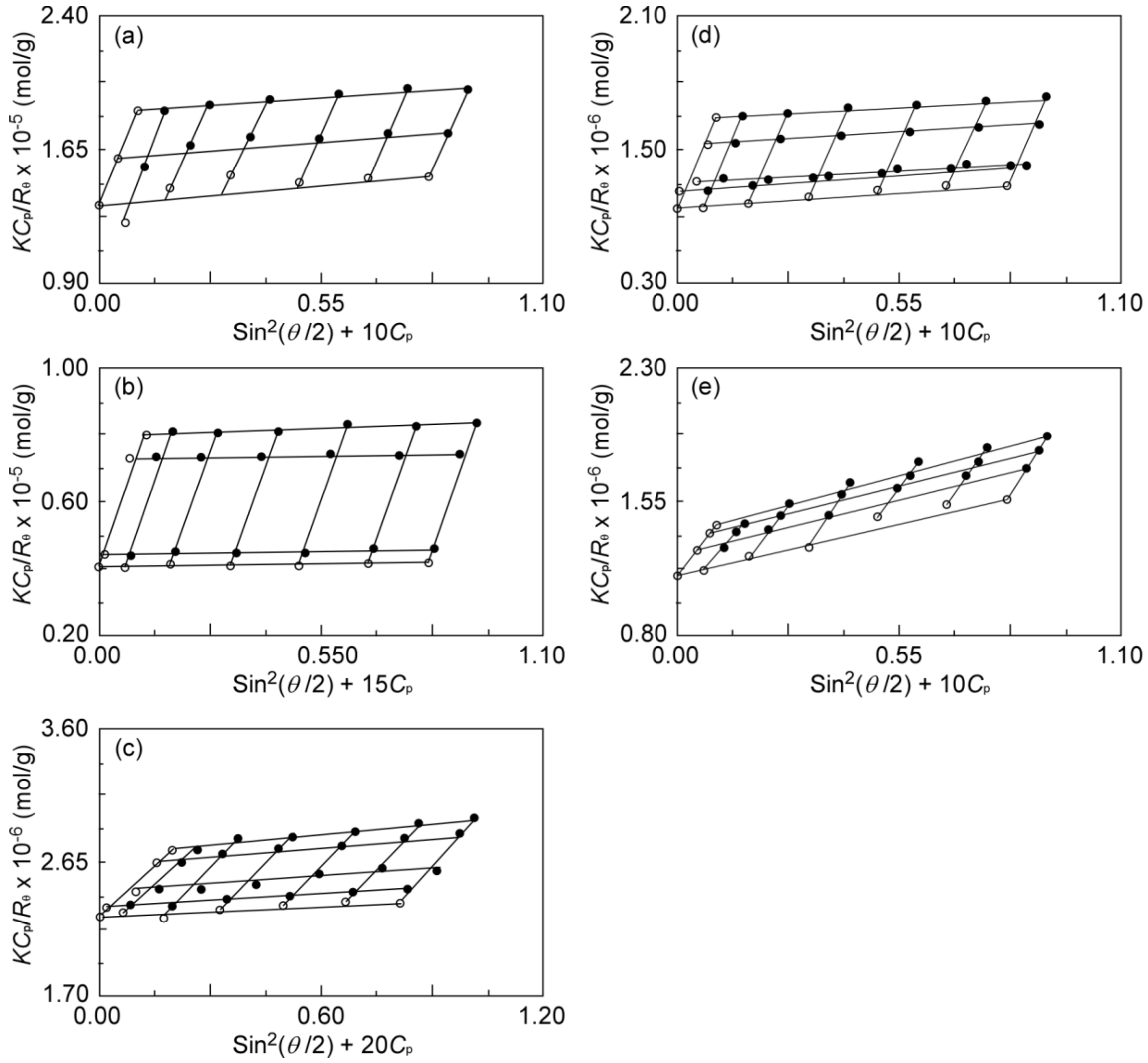

Figure S6. Zimm plots of P(MPC/DMA$_x$) in 0.1 M NaCl aqueous solutions where x = (a) 0, (b) 10, (c) 19, (d) 28, and (e) 38 mol%.

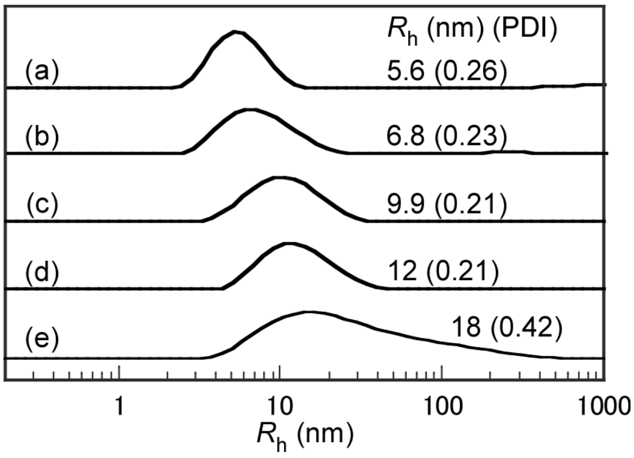

Figure S7. Hydrodynamic radius (R_h) distributions and polydispersity index (PDI) for P(MPC/DMA$_x$) in 0.1 M NaCl aqueous solutions at 25 °C where x = (a) 0, (b) 10, (c) 19, (d) 28, and (e) 38 mol%.

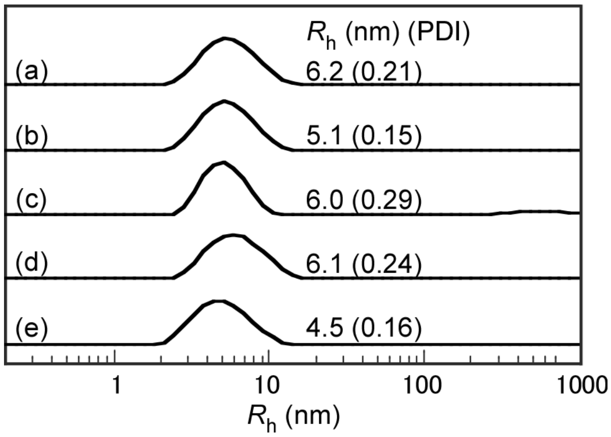

Figure S8. Hydrodynamic radius (R_h) distributions and polydispersity index (PDI) for P(MPC/DMA$_x$) in methanol at 25 °C where x = (a) 0, (b) 10, (c) 19, (d) 28, and (e) 38 mol%.

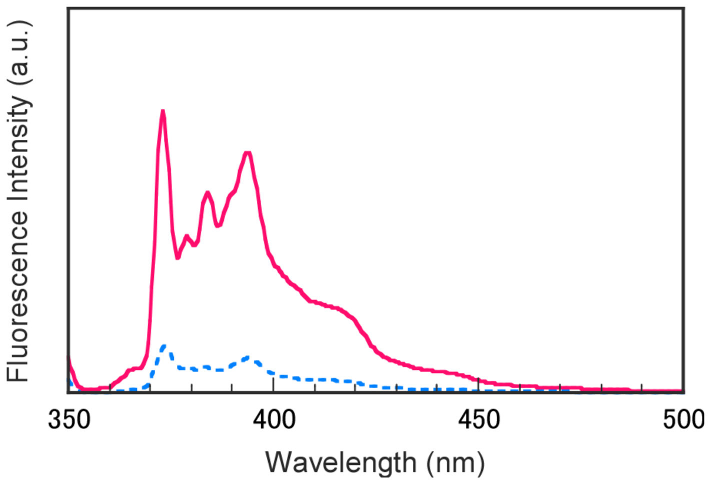

Figure S9. Fluorescence spectra of pyrene in the absence (---) and presence (—) of P(PMPC/DMA$_{38}$) in 0.1 M NaCl aqueous solutions excited at 334 nm. The excitation and emission slit widths were fixed at 20 and 5.0 nm, respectively.
